# A Relation between Obstructive Sleep Apnea in Pregnancy and Delivering Small for Gestational Age Infant—A Systematic Review

**DOI:** 10.3390/jcm12185972

**Published:** 2023-09-14

**Authors:** Alicja Grajczyk, Karolina Dżaman, Katarzyna Czerwaty, Monika Kasperczak, Magdalena Zgliczyńska, Anna Stępień, Katarzyna Kosińska-Kaczyńska

**Affiliations:** 1 Department of Otolaryngology, Centre of Postgraduate Medical Education, Marymoncka 99/103, 01-813 Warsaw, Poland; alicja.grajczyk@gmail.com (A.G.); katarzynaczerwaty@gmail.com (K.C.); a.e.serafin@gmail.com (A.S.); 2 Department of Obstetrics, Perinatology and Neonatology, Centre of Postgraduate Medical Education, Marymoncka 99/103, 01-813 Warsaw, Poland; monika.kasperczak@icloud.com (M.K.); zgliczynska.magda@gmail.com (M.Z.)

**Keywords:** small for gestational age, obstructive sleep apnea, pregnancy, upper airway resistance, sleep hypopnea, birth weight

## Abstract

Obstructive sleep apnea (OSA) during pregnancy can negatively affect both the mother and the baby. Our main goal is to show whether there is an association between OSA during pregnancy and delivering small for gestational age (SGA) infants. This systematic review was conducted according to the PRISMA 2020 statement using three databases: MEDLINE via PubMed, Scopus, and Cochrane Library. All databases were last accessed on 1 June 2023. The implemented systematic literature search identified 744 articles. After excluding reviews, meta-analyses, book chapters, case reports, and letters, 47 studies were analyzed, 18 of which finally met the inclusion criteria. The included studies mainly indicate that OSA during pregnancy may not significantly impact SGA, but some of them have shown the existence of this relation. Nevertheless, it is recommended that all pregnant women should be screened for symptoms of OSA and that sleep tests should be performed on those who show signs of it. Detecting and treating OSA early in pregnancy can help reduce the condition’s negative effects. However, more extensive studies are still needed to gather clear evidence on the impact of an OSA diagnosis on mothers and babies.

## 1. Introduction

Sleep is one of the essential biological functions of every human being. It is estimated that it constitutes about 30% of one’s lifetime. It is responsible for regeneration, maintaining health, and ensuring proper daily functioning. Sleep disturbances have been widely studied in pregnant women [1,2]. Although sleep disturbance does not have a strict definition, obstructive sleep apnea (OSA), snoring, inadequate sleep duration, poor sleep quality, insomnia symptoms, and restless legs syndrome can be included, and these symptoms may lead to various maternal complications and adverse fetal outcomes [2,3]. OSA, insomnia, and restless legs syndrome are among the most common sleep disorders during pregnancy [4]. Furthermore, sleep disturbances in pregnancy are associated with a history of premenstrual syndrome, hyperemesis gravidarum, vitamin B12 deficiency, and higher TSH levels [5]. In addition, the poor sleep quality observed in more than half of pregnant women may be due to pregnancy-related discomfort such as lower back pain, gastroesophageal reflux, increased urination, and difficulty changing position at night [6].

OSA is characterized by repetitive upper airway obstruction and significant reductions in airflow during sleep, which leads to repetitive hypoxia and fragmentation of rest. It is a common disorder in the general population, with a prevalence of 12% in US adults [7]. During pregnancy, specific physiological changes in the women’s body contribute to a higher prevalence of OSA. They include edema, increased body weight, reduced upper airway dimensions due to hyperemia of pregnancy, and increased upper airway collapsibility due to hormonal changes [8,9]. Airway closure increases during tidal ventilation, especially in the supine position. On the other hand, high levels of progesterone have a potentially protective effect because progesterone significantly upregulates ventilatory drive by influencing the central chemoreceptors [10]. Clinical factors associated with OSA in pregnancy include obesity, asthma, hypertension, and gestational diabetes mellitus [11,12,13,14,15,16], although the association between OSA and gestational diabetes mellitus is uncertain [17]. In addition, a higher risk of OSA is associated with pre-pregnancy BMI and Perceived Stress Scale score [18].

The diagnosis and definition of OSA are usually based on the American Academy of Sleep Medicine (AASM) recommendations [19]. The gold standard for OSA diagnosis is overnight polysomnography (PSG). It is a susceptible and specific method, however, not widely available. Therefore, research including large study groups screened via PSG is lacking. For this reason, the prevalence of OSA has also been examined based on subjective questionnaires like the Epworth Sleepiness Scale (ESS), Berlin Questionnaire (BQ), and STOP-Bang [20,21,22]. Unfortunately, the BQ has limited effectiveness in screening for OSA due to its predominant reliance on obesity identification [23]. On the other hand, STOP-Bang had positive predictive values of over 70% in the pregnant population [24] but had limited performance [25], and asking about snoring may be a more straightforward, effective predictor of OSA [26]. Another tool that may be applied to predicting OSA in pregnancy is the Sleep Apnea Symptom Score, which, when combined with patient characteristics, i.e., age, BMI, and partner-reported snoring and breathing pauses, shows reasonable sensitivity and specificity [27]. The pooled overall prevalence of OSA during pregnancy was 15% (95% CI 12–18%) [28], but it can reach even more than 40% in a pregnant population with significant obesity (BMI > 35 kg/m^2^) [29]. It should also be noted that the prevalence and severity of OSA deteriorate throughout pregnancy [30].

**Figure 1:**
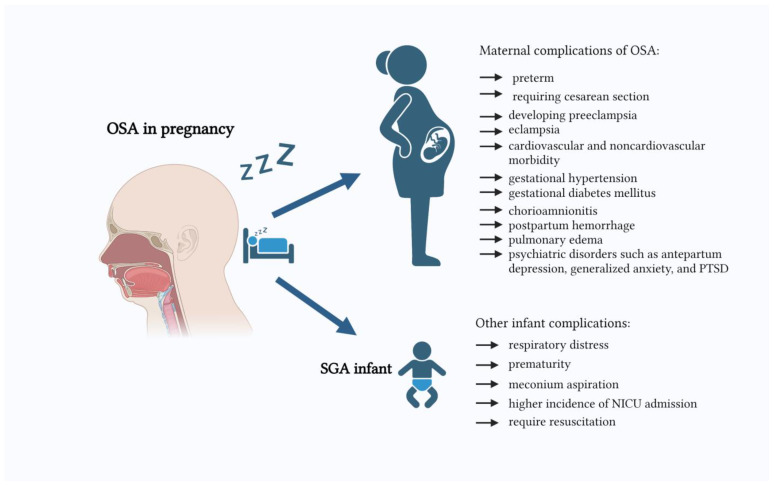
Maternal and infant complications of OSA in pregnancy.

Limited and conflicting data exist on the possible relationship between maternal sleep disturbances and perinatal outcomes (Figure 1). In animal studies, intermittent maternal hypoxia, a model of OSA, led to low birth weights (LBWs) of rat pups [31,32]. Moreover, the fluctuations in the oxygen partial pressure were translated from maternal to fetal blood, but transfer across the placenta reduced the magnitude of oxygenation fluctuations in a sheep model of gestational sleep apnea [33]. OSA is related to repetitive nocturnal oxygen desaturation, and the recurrent episodes of hypoxia and cortical arousal may lead to sympathetic activation and inflammation, subsequently leading to endothelial dysfunction. It results in placental dysfunction and pre-eclampsia development. An association between OSA and pre-eclampsia has been established [2,34]. However, placental dysfunction may also impair fetal growth [35]. Some authors report an increase in placental weight and obesity in newborns of mothers with OSA [36]. Therefore, there is still a clear need for a profound study of available data on the relationship between OSA and small gestational age infants (SGA) in pregnancy. Therefore, this present review was designed and conducted to investigate and understand the association between OSA and SGA.

## 2. Materials and Methods

### 2.1. Literature Search

This review was conducted according to the PRISMA 2020 statement [37]. The systematic literature search was completed using three databases: MEDLINE via PubMed, Scopus, and Cochrane Library. A detailed search design is shown in Table S1. We independently assessed its applicability by checking if it successfully covers three known articles on the chosen topic. All databases were last accessed on 1 June 2023.

**Figure 2:**
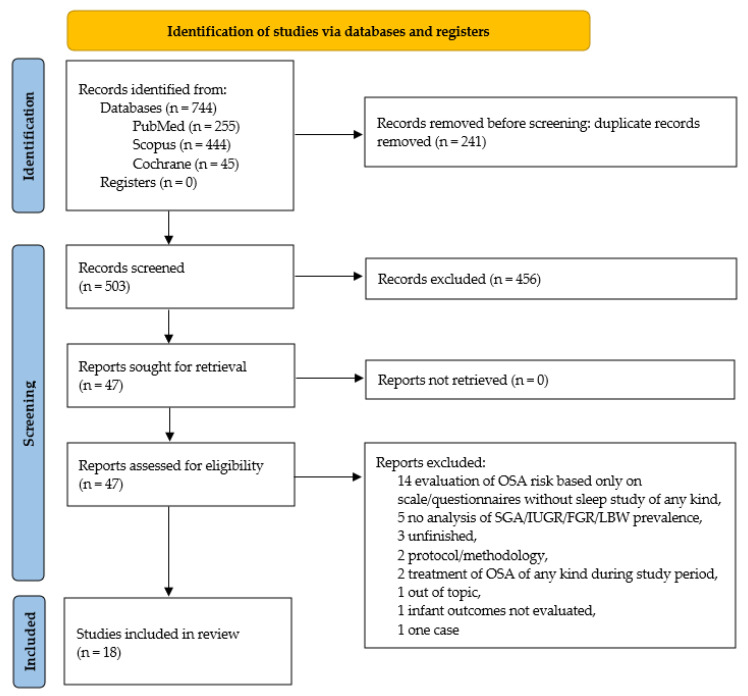
PRISMA 2020 flow diagram for new systematic reviews included searches of databases and registered only [37].

Two authors of this study have independently screened titles and abstracts of found studies. In the next step, full-text articles from 47 studies were assessed by two other authors for eligibility. Imposed inclusion and exclusion criteria are gathered in Table 1. The PRISMA Flowchart is shown in Figure 2.

As a result of the above-described process, 18 articles were finally included in this systematic review. We did not have to contact the authors of the retrieved studies to gain additional information. All disagreements between the study authors have been discussed in the presence of all authors, and a common consensus was established. 

### 2.2. Data Extraction

In the subsequent step, we designed and adapted to the topic’s original data extraction form and checked its relevancy on three randomly chosen studies. The research suitability was assessed, and the information from each study was obtained separately by two researchers. In cases of disagreement, conflicts were resolved through dialogue and agreement. We collected data on the authors, year, the origin of publication, main aim, sample characteristics, used diagnostic test, the acquired definition of OSA and fetal growth restriction (FGR), and the results. We did not perform any statistical synthesis of the results. This decision was mainly due to various study designs, adopted definitions, and measurement scales.

### 2.3. Risk of Bias Assessment

Two independent reviewers (A.G. and K.D.) assessed the risk of bias using the Newcastle–Ottawa quality assessment scale (NOS) for cohort and case–control studies [38]. The NOS for studies was used to assess study quality and any potential bias in the domains of subject selection, comparability, and the assessment of outcomes. Any inconsistencies in the ratings concerning the potential for bias or rationales behind such assessments were resolved through discussion to establish a mutual agreement between the two review authors. A total score of 0–3 was considered unsatisfactory, 4–5 points satisfactory, 6–7 points good, and 8–9 points very good (Table S2). Furthermore, the Discussion section outlines additional potential sources of bias that were not encompassed within this scale.

## 3. Results

### 3.1. Characteristics of the Included Studies

The implemented systematic literature search identified 744 articles. After adjusting for duplicates using EndNote X9 automatic duplicate search followed by manual verification, 503 studies remained. After excluding reviews, meta-analyses, book chapters, case reports, and letters, 47 studies were analyzed, 18 of which finally met the inclusion criteria. Details on the selection process are presented in a customized PRISMA flow chart in Figure 2. The primary characteristics of the studies included in this review are summarized in Table 2.

The NOS scores (Table S2) in all included studies ranged from 6 to 9 points, indicating a favorable level of methodological quality. These studies have demonstrated a strong commitment to addressing the critical aspects of study design, execution, and potential biases, resulting in findings that can be regarded with a high degree of confidence. The high-quality nature of these studies justifies their significance in contributing valuable insights to the field, yielding results that are not only reliable but also impactful.

We evaluated the included studies for quality of evidence and strength of recommendations using the GRADE approach. The presence of both high and moderate GRADE levels of evidence in most of our studies signifies a strong foundation for the conclusions drawn from these articles. Two studies have received a GRADE LOW level of evidence. However, it is crucial to emphasize that they play a pivotal role in contributing important insights and knowledge to the field.

### 3.2. Obstructive Sleep Apnea Diagnosis

#### 3.2.1. Preliminary Assessment of the Risk of Obstructive Sleep Apnea

Researchers often used eligible questionnaires to diagnose OSA in pregnancy, validated as a screening tool in non-pregnant populations. Most of the authors in this study utilized the BQ, which concentrated on self-reported snoring and apnea [40,42,43,49,50,55,56]. It has a sensitivity of 86% and a specificity of 77% in non-pregnant adults [22], while it only had a sensitivity and specificity of 35% and 63.8%, respectively, in pregnant women. Another questionnaire used to extract pregnant subjects suspected of apnea was the ESS, which measures daytime sleepiness [40,42,43,49,54,55,56]. In non-pregnant subjects, the ESS sensitivity was estimated to be from 66% to 93.5% and specificity from 48% to 100% [57,58]. However, in pregnancy, the ESS sensitivity and specificity are lower and assessed at a level of 36% and 77%, respectively.

Other researchers used the Multivariable Apnea Risk Index (MAP Index) [50] or the Pittsburgh Sleep Quality Index (PSQI) questionnaire [45]. The MAP Index, based on the frequency of self-reported symptoms of sleep-disordered breathing (SDB) and as a screening tool, characterizes a lack of specificity for OSA diagnosis [22]. The PSQI questionnaire consists of one part regarding self-reported sleep quality and the second part of bed-partner observation during the participant’s sleep. It should be noted that all the mentioned questionnaires are validated only in a non-pregnant population.

#### 3.2.2. Objective Methods of Diagnosing Obstructive Sleep Apnea

The diagnosis of OSA requires an objective assessment of respiratory events using a broad range of devices. The AASM categorized these tools as type I, II, III, or IV. The gold standard for OSA diagnosis is attended PSG (type I), which involves a collection of at least seven data channels, including electroencephalogram (EEG) and electrooculogram for sleep staging, electromyogram, electrocardiogram, and respiratory channels. Some authors followed this direction and performed PSG in the third trimester of pregnancy [42,55,56].

Unattended studies are called home sleep apnea testing (HSAT) and referred by the traditional type II to type IV classification system. Type II studies use full PSG but are unattended and thus can be performed outside the hospital. Some researchers performed PSG in a pregnant woman’s home, others [50] in the hospital or at home, depending on the subject’s preferences. Moreover, the retrospective studies analyzed in this paper did not specify whether PSG was carried out in the hospital or at home [44,46,52,53].

Type III studies use modified HSAT measuring a minimum of four channels, including ventilation (at least two channels of respiratory movement or a combination of respiratory movement and airflow), heart rate, electrocardiography, and oxygen saturation. Hawkins et al. used a type III device twice during pregnancy (the first: between 6 and 15 weeks of gestation; the second: between 22 and 31 weeks of gestation) to assess prevalence at the beginning and in advanced gravity. Louis et al. [51] utilized type III HSAT to test pregnant participants with a high risk of OSA, but all subjects diagnosed with OSA underwent attended PSG ultimately. Wilson et al. [39] and Antony et al. [49] examined participants using type I or type III HSAT, but Antony et al. assessed subjects regardless of the device used in the hospital. In contrast, Wilson et al. assessed women depending on the methods in a hospital (type I) or home (type III). Referring to new technologies, devices using peripheral arterial tonometry (PAT) were also classified as type III (Watch-PAT 100, Watch-PAT 200). Some investigators [48] used Watch-PAT 100 twice during pregnancy (the first between 6 and 20 weeks of gestation; the second between 28 and 37 weeks of gestation), while others [43] utilized the Watch-PAT200 only one time during gravity between 33 and 36 weeks of pregnancy.

The last category, type IV HSAT, included unattended, single-, or dual-parameter recording, typically oxygen saturation and/or heart rate, or in some cases, just airflow. Yin et al. [54] assessed pregnant women using single-night saturation monitoring in/outside of the hospital.

### 3.3. Obstructive Sleep Apnea Definition

The studies analyzed in this systematic review are incoherent according to the definitions of the breathing events and criteria of OSA diagnosis. Moreover, some authors used the terms SDB and OSA synonymously. Few investigators [46,52] could not write the specifications because of the retrospective character of their papers. Others [43,44] defined OSA as at least five or more apnea–hypopnea indices (AHI) per hour of sleep but did not have precise definitions of apnea and hypopnea. But the next ones [54] counted the number of events when oxygen saturation decreased by more than 4% below the baseline saturation and expressed it as the number of 4% dips per hour. Patients met the criteria of OSA when five or more such dips per hour were estimated. Facco et al. [48] scored an AHI event based on whether a PAT amplitude reduction occurred with 3% or more significant desaturation or 4% or greater desaturation occurred and defined SDB when AHI was five or more per hour. On the other hand, in another study [49], OSA was diagnosed with RDI ≥ 5/h in the subset of PSG and with AHI ≥ 5/h in the subset of T3D. The author measured breathing events according to the AASM Manual for Scoring Sleep and Associated Events, 2007, but did not specify which of the two definitions of hypopnea was used to evaluate PSG. Wilson et al. [39] also followed the rules included in the AASM statement, but the definition of hypopnea revealed in the study (decrease in airflow ≥30% from the baseline for ≥10 s and followed by either an oxygen desaturation of ≥3% or an EEG cortical arousal) was incoherent with those in the statement. In the first analysis, SDB was defined as RDI ≥ 5/h, with a secondary analysis performed with SDB defined as RDI ≥ 15/h. Otherwise, Suri et al. [42] scored the events as apnea when the drop was >90% of the baseline lasting for ≥10 s, and hypopnea required a ≥30% reduction in airflow for ≥10 s and was accompanied by a ≥3% oxygen desaturation. SDB criteria required AHI ≥ 5 and symptoms such as excessive daytime sleepiness or AHI > 15 with or without associated symptoms. Interestingly, some authors [45] estimated respiratory events using standard Chicago criteria (hypopnea is defined as >50% reduction in a measure of airflow or a clear reduction in airflow accompanied by arousal or a 3% oxygen desaturation lasting at least 10 s) and put the diagnose with SDB when AHI ≥ 5/h. The other researchers assumed that apnea complete cessation in a valid measure of airflow going on ≥10 s. However, the definition of hypopnea differed from each other. Koken et al. [56] and Sahin et al. [55] counted the events with an airflow decrease of ≥50% accompanied by a ≥3% desaturation in the preceding 30 s and a reduction in chest wall movement and/or arousal. Surprisingly, Louis et al. used two different definitions of hypopnea depending on the year of research publication. In 2010, they used the definition of hypopnea as a reduction of ≥10 s in airflow (≥30%) accompanied by either arousal or ≥3% desaturation [53]. But in another study [51], hypopnea was defined as a ≥50% reduction in airflow with a ≥3% decrease in oxygen saturation. The last author [50] scored the breathing events with a decrease in airflow ≥10 s associated with ≥3% desaturation or arousal. 

Moreover, some authors [51,53,55] put down a diagnosis of OSA if AHI ≥ 5/h, the next [50] when RDI ≥ 5/h, while others [56] noted the diagnosis when RDI ≥ 10/h or RDI ≥ 5/h with the lowest nocturnal oxyhemoglobin desaturation of ≥90%.

To sum up, incoherence in studies regarding breathing events and the criteria for OSA diagnosis can lead to confusion and inconsistency in the literature. Having clear definitions and criteria is important to ensure accurate and standardized research findings. 

### 3.4. Relation between Obstructive Sleep Apnea and Small Gestational Age

All 18 included studies investigated the association between OSA in pregnancy and fetal growth, and the results of each study are presented in Table 2.

Several studies [39,44,45,50,52] have observed a relationship between OSA and SGA or fetal growth retardation. In a study by Chen et al. [52], women diagnosed with OSA experienced statistically higher rates of adverse outcomes that included LBW infants (8.6% vs. 4.2%, *p* < 0.001) and SGA infants (18.3% vs. 13.5%, *p* < 0.001) than those without OSA. Pamid obtained similar results in a prospective study [45] on a relatively healthy and non-obese group, where a significant association was found between a diagnosis of SDB based on PSG in the third trimester of pregnancy and an increased likelihood of delivering an SGA baby (*p* < 0.05, OR 2.57–3.07, depending on the AHI thresholds used). Moreover, other PSG-based SDB indicators, such as the obstructive hypopnea index and the oxygen desaturation index, consistently demonstrated an increased probability of having an SGA baby [45]. Furthermore, some studies showed a correlation between OSA decrease and fetal weight centiles in the third trimester [44,50]. Although there were no notable disparities in the percentage of infants with a birth weight below the 10th percentile between women with and without OSA (23% vs. 25%, *p* = 1.0), significant differences were found in the proportion of infants experiencing a deceleration in fetal growth during the final trimester (61% vs. 29%, *p* = 0.0095). In total, 61% of women with untreated OSA and 35% without OSA exhibited impaired fetal growth, indicated by either a birth weight below the 10th percentile or a deceleration in fetal growth (*p* = 0.043) [44].

However, some authors found an association between OSA and SGA in hypertensive women but not in normotensive women [39]. Additionally, mild SDB did not result in worse acute or long-term fetal outcomes in women with hypertensive disorders of pregnancy or in BMI-matched normotensive controls. Surprisingly, the presence of SDB in hypertensive disorders of pregnancy cases was associated with improved fetal growth outcomes, indicating a better prognosis [39]. Moreover, it was observed that women with restricted growth had a higher tendency to fall asleep on their backs than those with normally developed fetuses (36.6% vs. 7.5%, *p* < 0.001). Sleeping on the back was associated with FGR and birthweight at delivery, although a causal relationship cannot be established. The information presented in the study [39] was derived from three separate studies that followed group participants over time to examine SDB in different pregnancy scenarios. The first study focused on pregnant women without underlying health conditions [16]. The second study examined pregnancies complicated by FGR, comparing them to control groups with similar BMI and gestation periods [19]. Finally, the third study investigated hypertensive disorders of pregnancy, including gestational hypertension, pre-eclampsia, and chronic hypertension, again comparing them to control groups with matched BMI and gestation periods [20]. FGR was determined using the ultrasound Delphi consensus criteria [23], which consider parameters such as estimated fetal weight, abdominal circumference falling below the 3rd centile, estimated fetal weight, or abdominal circumference below the 10th centile accompanied by abnormal fetoplacental Doppler findings on ultrasound. It was observed that women who had fetuses with growth restriction had a significantly higher tendency to initiate and maintain a supine sleep position throughout the night compared to women with healthy pregnancies. Moreover, a preference for sleeping in the supine position was linked to lower birthweight in infants. However, it should be noted that another study found that the supine position was associated with more respiratory events and deeper oxygen desaturation in pregnant women with OSA. Still, no effect on perinatal outcomes was observed [59]. Finally, positional therapy can significantly reduce the percentage of sleep time in the supine position during late pregnancy [60].

On the other hand, there were also studies [40,43,53,54,55,56] where no relationship between OSA during pregnancy and SGA was observed. Additionally, Kőken et al. [56] observed no significant differences in IUGR prevalence, average infant birth weight, and incidence of complications during birth between infants born to mothers who reported snoring and those born to mothers who did not. In two other prospective studies [40,43], the diagnosis of OSA was based on PSG in an average population of pregnant women and found no association between OSA and SGA.

In the studies analyzed above, an association between OSA and SGA or no correlation between these factors was observed. However, interestingly, there are also studies [43,46] in which the opposite results were obtained, and a relation between OSA and large newborn gestational age (LGA) was found. In a study by Bin et al. [46], sleep apnea showed a significant association with LGA (RR 1.27; CI 1.04–1.55), as well as significant associations with various pregnancy outcomes, such as a 5 min Apgar score of less than 7 (RR 1.60; CI 1.07–2.38), which was also found in a study by Chen et al. [52]. Telerant’s study [43] is the first to show that mild maternal OSA in non-obese pregnant women without gestational diabetes, or hypertensive disorders is associated with accelerated fetal growth, manifested by various growth parameters such as weight, length, and adiposity, as well as an increased risk of LGA. In a cohort of non-obese women without pregnancy complications, the effect of mild OSA on fetal growth was investigated while minimizing potential confounders such as maternal obesity and adverse pregnancy complications that could potentially affect the fetus [43].

To sum up, there are many inconsistencies regarding the effect of OSA on SGA and fetal growth. Some researchers confirmed a positive correlation between OSA in pregnancy and fetal growth retardation. Others noticed that relationships were only in hypertensive women. Some researchers did not observe any association between those two events. Interestingly, there are also reports in contrast where a relation between OSA and LGA was found.

### 3.5. Limitations of the Included Studies

The mean limitation of the included studies is the variety of OSA diagnosis tools and impaired fetal growth definition. Several differences in SGA diagnosis were also found among the included studies. In three studies, the SGA definition was unavailable [42,51,53], while in a study by Louis et al. [47], poor fetal growth code in hospital records was the only criterion. Most studies used the SGA definition of birthweight below the 10th percentile for gestational age. However, four customized growth charts were used [39,44,45,50]. Yin et al. [54] used abdominal circumference measure <10th centile for gestational age, while Facco et al. [48] used birthweight <5% for gestational age. Three studies included additional analysis of slowing fetal third-trimester growth, defining it as a fall in customized percentile >33% between the scan and delivery [39,44,50]. Various definitions of SGA contribute to bias.

## 4. Discussion

Studies have investigated the relationship between OSA and SGA, and their results have been conflicting [61]. This may relate to differences in OSA diagnosis, usually based on PSG in small sample-sized studies or on subjective questionnaires and national datasets in large cohorts. Two recent meta-analyses included studies with various diagnostic methods, also based on non-objective methods and heterogenous populations, and concluded them jointly [2,28].

Some investigators suggest that OSA during pregnancy can lead to unfavorable maternal and fetal outcomes, which may be even more relevant in multiple pregnancies [62]. Therefore, screening all pregnant women for OSA symptoms and offering PSG to suspected cases and those with high-risk pregnancies, excessive daytime sleepiness, chronic hypertension, or obesity is recommended [14,63,64,65,66,67,68]. Additionally, BMI, age, and the presence of tongue enlargement can be used to predict the risk of OSA in pregnancy [69]. Moreover, Mallampati class and upright neck circumference were proposed to predict sleep-disordered breathing in pregnancy [70].

There are differences in when to screen for OSA in pregnant women. Some authors argue that patients should be screened at 24–35 weeks of pregnancy because of the higher incidence of OSA in the middle and third trimesters of pregnancy [71], but others believe that screening is better conducted earlier because of the decline in sleep quality during pregnancy and the inadequate test result associated with the discomfort of sleeping with a monitoring device [72,73]. The early detection and management of OSA during pregnancy may reduce the adverse outcomes of the condition. Additionally, in some cases, a single sleep study during pregnancy may not be sufficient to diagnose correctly [74]. Unfortunately, the percentage of pregnant patients who complete OSA testing among those referred for it is also unsatisfactory [75].

The use of continuous positive airway pressure (CPAP) in pregnancy, which has been shown to have anti-inflammatory properties and decrease oxidative stress [76], can have a positive effect on hypertension in pregnant women, preterm labor, birth weight increment, a higher Apgar, or a reduction in serum uric acid and tumor necrosis factor-alpha [77,78,79,80,81]. CPAP treatment in women with gestational diabetes and OSA improved insulin secretion but not glucose levels [82]. In addition to CPAP treatment, various methods of treating OSA, such as mandibular advancement splints [83], have been studied in a population of pregnant women.

Significant limitations occurred in most of the studies included in the systematic review. Five studies included the usual cohort of pregnant women and used PSG for OSA diagnosis. In four of them, no association between OSA and SGA was found [40,43,44,45,50]. A prospective study by Pamidi et al. [45] revealed a relationship between OSA and delivering SGA infants. However, the result was on the verge of statistical significance.

Apart from SGA, the relationship between OSA and other aspects of fetal growth was analyzed. Slowing fetal third-trimester growth was investigated and confirmed in women with OSA [50]. On the other hand, some researchers [40,43] reported a higher risk of LGA infants in women suffering from sleep-disordered breathing. Therefore, there is still a clear need for a profound study of the available data on the relationship between OSA and SGA in pregnancy.

Different components of OSA (like hypoxia or sleep fragmentation) or the severity of OSA may have a varying impact on fetal growth. OSA may influence maternal lipid levels and fetal insulin. The metabolic consequences of OSA or its specific aspects might contribute to a metabolic milieu favorable for accelerated fetal growth.

Studies have several limitations that require consideration. Cohorts included only a limited number of women with severe OSA and were limited to obese patients. Moreover, there are different OSA and FGR definitions in those studies. The next one is unable to gather data on the deliveries of some patients who transferred their care out of the system, mainly from the control group. This may result in overestimation if those women also had severe outcomes. Additionally, women diagnosed with OSA had other medical conditions like hypertension and diabetes, which must be taken into consideration. Other limitations are various methods of OSA determination, like PSG, in-home portable PSG, self-reported ESS, nocturnal pulse oximetry, etc.

## 5. Conclusions

OSA can lead to adverse maternal and neonatal outcomes regardless of other risk factors, highlighting the importance of identifying and treating OSA in pregnant women via improved screening methods and treatments. In some studies, we found a relation between OSA and SGA, but we also know that other factors impact study results. Women with multiple comorbid conditions were potentially more likely to be screened and diagnosed with OSA. 

Physicians and midwives managing pregnancies should pay special attention to whether pregnant women have risk factors and symptoms of OSA and should include this information in their interviews with patients at the first visit. Pregnant women should be screened for OSA, and an increased level of health care should be provided to this group. Women at risk or with symptoms of OSA should be offered PSG. Given that obesity is a risk factor for OSA, obese women should be referred to a nutritionist to help treat obesity, if possible, even before pregnancy. In the case of OSA in pregnancy, early diagnosis is very important so that treatment can be quickly instituted and the adverse effects of this disease on patients and their children can be reduced. Some consequences of OSA in pregnancy may be irreversible if the treatment is delayed. It is necessary to improve screening methods and increase the availability of treatment for OSA in pregnancy.

To summarize, larger randomized controlled trials are necessary to establish conclusive evidence of the maternal and fetal outcomes related to OSA diagnosis during pregnancy. Additional research is required to identify and understand the potential impact of OSA-induced biochemical, metabolic, and immune alterations that occur in OSA and understand their impact on maternal and fetal outcomes. Planned studies should focus on eliminating the influence of other factors that may affect outcomes, such as comorbidities like hypertension, diabetes, or other diseases that may result in maternal hypoxia, as well as the influence of weight, age of maternal socioeconomic status, and smoking during pregnancy. Control groups should be matched to study groups that account for the above factors. In particular, the co-occurrence of diabetes and obesity among pregnant women may have a key impact on the birth weight of fetuses of mothers with OSA. It is also important to have homogeneous groups of patients concerning the severity of OSA to study the relationship between the severity of OSA and the occurrence of complications in mothers and fetuses. There is still a need for research on the effect of OSA treatment in pregnancy on reducing the negative effects of the disease, as previous studies of the efficacy and safety of CPAP in pregnancy have been conducted on small groups of patients, so whether short-term CPAP treatment affects fetal well-being has not been sufficiently investigated. 

## Figures and Tables

**Table 1:** Inclusion and exclusion criteria.

Inclusion Criteria	Exclusion Criteria
Studies on the impact of OSA on the prevalence of SGA/IUGR/FGR/LBW in human pregnancies	Studies on another topic (including studies on the association between OSA and birthweight NOT reporting the prevalence of SGA/IUGR/LBW) or animal studies
OSA was diagnosed based on the examination of sleep of any kind (for example, PSG, watch pat home sleep test)	OSA was only suspected, based on, for example, scales such as STOP-Bang or BQ
Language: English	Language other than English
Original studies	Reviews and meta-analyses
	Case reports
	Editorials/Letters/Commentaries
	Book chapters
	Protocols
Finished study	Unfinished study

BQ—Berlin Questionnaire; FGR—fetal growth restriction; IUGR—intrauterine growth restriction; LBW—low birthweight; OSA—obstructive sleep apnea; PSG—polysomnography; SGA—small for gestational age.

**Table 2:** Basic characteristics of the studies included in the systematic review.

Study	Main Aims of the Study	Study Design	Population	Diagnostic Test for OSA	OSA Definition	FGR Definition	Results	GRADE *
Wilson et al. (2022) [39]	To examine the distribution of sleep positions throughout the night and the associations between sleeping in the supine position, SDB, and various outcomes related to pregnancy	Three prospective cohort studies investigating SDB	187 pregnant women (26 pregnancies with FGR, 15 pregnancies with FGR and a hypertensive disorder)	Overnight PSG	RDI ≥ 5/h AHI ≥ 5/h	The ultrasound Delphi consensus criteria were utilized to define FGR	Women who had fetuses with growth restriction exhibited a significantly higher tendency to initiate sleep in a supine position and remain in that position throughout the night in comparison to women with uncomplicated pregnancies; a predilection for sleeping on the back was linked to lower birthweight in infants	H
Hawkins et al. (2021) [40]	To determine if objectively measured SDB in pregnancy is associated with infant birthweight	Prospective cohort study	2.211 nulliparous women	Overnight sleep assessment with the Embletta Gold monitor, a self-administered Level 3 in-home sleep apnea monitor	AHI ≥ 5/h	Birthweight < 10th percentile for gestational age	Early pregnancy SDB, mid-pregnancy SDB, or SDB were not associated with SGA in unadjusted or adjusted models	H
Wilson et al. (2020) [41]	To determine whether the presence of co-existing SDB is associated with worse perinatal outcomes among women diagnosed with hypertensive disorder of pregnancy compared with normotensive controls	Prospective case–control study	40 pregnant women with gestational hypertension or pre-eclampsia not requiring immediate delivery between 26 and 37 weeks gestation and 42 control participants with normotensive uncomplicated pregnancies one-to-one matched by BMI	Overnight PSG	AHI ≥ 5/h	Birthweight < 10th percentile for gestational age	FGR: hypertension group: FGR at birth 6 (30.0%) SDB vs. 13 (68.4%) no SDB, *p* = 0.026; no hypertension group: no differences between SDB and no SDB; impaired fetal growth: hypertension group: 10 (50%) vs. 15 (78.9%), = = 0.18, respectively; no hypertension group: 4 (28.6%) vs. 5 (19.2%), *p* = 0.69, respectively	M
Suri et al. (2019) [42]	To investigate if SDB correlates with adverse pregnancy outcomes in hypertensive women	Prospective case–control study	40 women with new-onset hypertension of pregnancy and 60 age-matched normotensive pregnant women	Overnight PSG	AHI ≥ 5/h, along with symptoms such as excessive daytime sleepiness or an AHI > 15 with or without associated symptoms	Not specified	Hypertension group: higher mean AHI in the IUGR group (13.168 ± 3.5015 vs. 9.786 ± 3.0630, *p* < 0.001) control group: higher mean AHI in the IUGR group (9.8 ± 3.9 vs. 6.8 ± 1.4, *p* = 0.002)	M
Telerant et al. (2018) [43]	To investigate the association between maternal OSA and fetal growth	Prospective cohort study	155 non-obese women in the third trimester of a singleton, uncomplicated pregnancy	Overnight sleep study between 33 and 36 weeks of gestation with watch-PAT 200 device	AHI > 5/h	Birthweight < 10th percentile for gestational age	No differences in SGA rates	H
Kneitel et al. (2018) [44]	Whether maternal OSA is associated with changes in the fetal growth trajectory	Retrospective study	79 pregnant women in singleton pregnancies: 48 without and 31 with OSA	Overnight PSG	AHI ≥ 5/h	Customized birth weight < 10th centile or a slowing in customized estimated fetal weight centile by >33% in the third trimester	Birth weight < 10th centile: 23% vs. 25%, *p* = 1.0; impaired fetal growth: 61% vs. 35%, *p* = 0.043; impaired fetal growth: aOR 3.4, 95% CI: 1.2–9.9; fall in fetal growth centile by >33%: aOR 4.3, 95% CI: 1.3–12.8	H
Pamidi et al. (2016) [45]	To examine whether a PSG-based diagnosis of SDB in the third trimester is associated with the delivery of SGA infants	Prospective cohort study	234 women in a singleton pregnancy, with a 3rd-trimester scan estimated fetal weight < 75 percentile	Overnight PSG	AHI ≥ 5/h (cut-offs of 5/h, 10/h, 15/h and 30/h)	Birthweight < 10th percentile for gestational age	AHI ≥ 5/h: OR 3.07 (95% CI 1.01–9.26); AHI ≥ 10/h: OR 2.65 (1.15–6.1); AHI ≥ 15/h: OR 2.57 (1.02–6.48)	H
Bin et al. (2016) [46]	To examine the association between sleep apnea and pregnancy outcomes	Population-based cohort study	Data from two sets of routinely collected health data: the NSW Perinatal Data Collection (birth records) and the NSW Admitted Patient Data Collection (hospital records): 636,227 women	Not specified	Women with a sleep apnea code in hospital records in the year before or during pregnancy	Birthweight < 10th percentile for gestational age and sex	SGA: RR 0.77 (95% CO: 0.58–1.03) Adjusted RR 0.81 (95% CI: 0.61–1.08)	L
Louis et al. (2014) [47]	To examine associations between OSA and severe clinical and pregnancy-related morbidities	Retrospective, cross-sectional analysis	55,781,965 pregnancy-related inpatient hospital discharges from 1998–2009 from the Nationwide Inpatient Sample (NIS), the largest all-payer, publicly available inpatient database in the United States	Not specified	Women with a sleep apnea code in hospital records	Poor fetal growth code in hospital records	Poor fetal growth OR 1.28 (1.02–1.62) (adjustment for maternal age; race/ethnicity; household income; multiple births; tobacco, alcohol, and drug use; primary payer; and rural/urban status; maternal obesity); OR 1.21 (0.96–1.53) (additional adjustment for clinical comorbidities (coronary heart disease, anemia, hyperlipidemia, hypothyroidism, disorders of the adrenal gland, pre-pregnancy diabetes, and pre-pregnancy hypertension)	L
Facco et al. (2014) [48]	To examine the relationship between SDB and adverse pregnancy outcomes in a high-risk cohort	Planned secondary analysis of a study designed to evaluate the trends in SDB across pregnancy among women at high risk for developing pre-eclampsia	128 women with pre-pregnancy BMI ≥ 30 kg/m^2^, chronic hypertension, pregestational diabetes (type 1 or type 2), a prior history of pre-eclampsia and/or a twin gestation	Overnight sleep evaluation with the Watch- PAT100 during early pregnancy (between 6 and 20 weeks of gestation) and late pregnancy (between 28 and 37 weeks of gestation)	AHI ≥ 5/h	Birthweight < 5% for gestational age	No association between SDB and birthweight < 5% for gestational age	H
Antony et al. (2014) [49]	To evaluate if OSA is associated with SGA after stratifying by obesity status	Prospective observational study	58 pregnant women	Overnight PSG	RDI > 5	Birthweight <10th percentile for gestational age	No association between SGA and snoring or BQ positive result	H
Fung et al. (2013) [50]	To determine whether OSA is associated with reduced fetal growth	Prospective longitudinal study	41 pregnant women in singleton uncomplicated pregnancy	Overnight PSG	RDI ≥ 5	Birthweight < 10th percentile for gestational age or a slowing in customized estimated fetal weight centile by >33% in the third trimester	Birthweight < 10th centile: 2/14 vs. 3/27 controls, *p* = 1; slowing third trimester growth: 4/14 vs. 0/27 controls, *p* < 0.001	H
Louis et al. (2012) [51]	To identify the risk factors for OSA and to describe the pregnancy outcome in obese women with OSA	Prospective observational study	158 obese pregnant women enrolled in Sleep Apnea in Pregnancy Screening Study (SAPSS)	In-home portable PSG with the ARES Unicorder 5.2 (B-Alert, Carlsbad, CA)	AHI ≥ 5/h	Not specified	No differences in birthweight between the OSA and control groups; 6 neonates were classified as SGA (1 in the OSA group and 5 in the control group)	M
Chen et al. (2012) [52]	To estimate the risk of adverse pregnancy outcomes in OSA	Retrospective analysis of 2 nationwide, population-based datasets	791 women who received a diagnosis of OSA and 3955 randomly selected women without OSA	PSG during ambulatory care visits within 1 year before their index deliveries	OSA diagnosis extracted from the Taiwan National Health Insurance Research Dataset	LBW < 2500 g; SGA <10th percentile for gestational age	Women with OSA had higher prevalence of LBW infants (8.6% vs. 4.2%, *p* = 0.001) and SGA infants (18.3% vs. 13.5%, *p* = 0.001); LBW: aOR 1.76 (95% CI: 1.28–2.40) SGA: aOR 1.34 (95% CI: 1.09–1.66)	H
Louis et al. (2010) [53]	To estimate the maternal and neonatal morbidities associated with OSA in pregnancy	Retrospective cohort study	57 women with OSA versus 114 normal-weight and obese randomly selected controls	Standard 15-channel PSG	AHI ≥ 5/h	Not specified	No differences in SGA occurrence between the groups (4 cases of SGA neonates in the OSA group, 8 cases of SGA in the obese group, and 11 cases of SGA in the normal-weight group)	H
Yin et al. (2008) [54]	To evaluate if some cases of IUGR or pre-eclampsia may be caused by unrecognized OSA in the mother	Prospective case–control study	178 women in the third trimester of pregnancy: 78 normal pregnant, 18 with hypertension, 25 with PE, 46 with IUGR, 8 with PE+IUGR, 3 with hypertension+IUGR	Self-reported ESS and nocturnal pulse oximetry	≥5 oxygen desaturation events (oxygen saturation decrease >4% below the baseline saturation)	Abdominal circumference measure <10th centile for gestational age	OSA in 0/57 (95% CI: 0–6.3%) among patients with IUGR	M
Sahin et al. (2007) [55]	To observe nonstress test changes during maternal desaturation and assess maternal and fetal outcomes	Prospective observational study	35 pregnant women with self-reported frequent snoring or any degree of apnea, according to the BQ	Overnight PSG	AHI ≥ 5/h	Birthweight < 10th percentile for gestational age	OSA in 4 (11.4%) of 35 pregnant women who underwent PSG, 3 of whom (75%) had fetal heart decelerations accompanying maternal desaturation; the neonates of women diagnosed with OSA had lower mean Apgar scores and birth weights compared to neonates of women without OSA	M
Kőken et al. (2007) [56]	To determine the levels of oxidative stress markers in pregnant women who snore	Prospective case control study	40 snoring and 43 non-snoring women in the second or third trimester of pregnancy	Overnight PSG	RDI ≥ 10/h or ≥5/h with the lowest nocturnal oxyhemoglobin desaturation of 90% or less	Birthweight < 10th percentile for gestational age	No differences in birthweight; 1 case of IUGR in a non-snoring group, none in the snoring group	M

95% CI—95% confidence interval; AHI—Apnea-Hypopnea Index; aOR—adjusted odds ratio; BQ—Berlin Questionnaire; ESS—Epworth Sleepiness Scale; FGR—fetal growth restriction; IUGR—intrauterine growth restriction; OSA—obstructive sleep apnea; LBW—low birthweight; PSG—polysomnography; RDI—respiratory disturbance index [RDI is the number of apneas (cessation of airflow ≥ 10 s), hypopneas (reduction in airflow ≥ 10 s associated with an oxygen desaturation of ≥3% or an arousal) and respiratory event-related arousals (sequence of breaths lasting ≥ 10 s associated with flattening of the nasal pressure waveform leading to an arousal from sleep) recorded per hour of sleep]; RR—risk ratio; SDB—sleep-disordered breathing; SGA—small for gestational age; * Assessment of the quality of evidence and strength of recommendations using the GRADE approach: L—low, M—moderate, H—high (no included study was rated “very low”).

## Data Availability

Not applicable.

## Supplementary Materials

The following supporting information can be downloaded at: https://www.mdpi.com/article/10.3390/jcm12185972/s1, Table S1: Search strategy, Table S2: Risk of bias assessed using the Newcastle-Ottawa quality assessment scale for cohort and case–control studies. A total score of 0–3 was considered unsatisfactory, 4–5 points satisfactory, 6–7 13 points good, and 8–9 points very good.

## References

[1] Middleton P.G. (2022). Obstructive sleep apnoea and sleep disorders in pregnancy. Best Pract. Res. Clin. Obstet. Gynaecol..

[2] Yang Z., Zhu Z., Wang C., Zhang F., Zeng H. (2022). Association between adverse perinatal outcomes and sleep disturbances during pregnancy: A systematic review and meta-analysis. J. Matern.-Fetal Neonatal Med..

[3] Lu Q., Zhang X., Wang Y., Li J., Xu Y., Song X., Su S., Zhu X., Vitiello M.V., Shi J. (2021). Sleep disturbances during pregnancy and adverse maternal and fetal outcomes: A systematic review and meta-analysis. Sleep Med. Rev..

[4] Facco F.L., Chan M., Patel S.R. (2022). Common Sleep Disorders in Pregnancy. Obstet. Gynecol..

[5] Kadıoğlu N., Sert U.Y., Sariaslan S.G., Mursel K., Celen S. (2022). Sleep Disorders in Pregnancy, Influencing Factors and Quality of Life. Z. Geburtshilfe Neonatol..

[6] Silvestri R., Aricò I. (2019). Sleep disorders in pregnancy. Sleep Sci..

[7] American Academy of Sleep Medicine (2016). Hidden Health Crisis Costing America Billions: Underdiagnosing and Undertreating Obstructive Sleep Apnea Draining Healthcare System.

[8] Edwards N., Middleton P., Blyton D., Sullivan C. (2002). Sleep disordered breathing and pregnancy. Thorax.

[9] Konstanty-Kurkiewicz V., Dzięciołowska-Baran E., Szczurowski J., Gawlikowska-Sroka A. (2022). Sleep-Disordered Breathing in Pregnancy. Adv. Exp. Med. Biol..

[10] White D.P., Douglas N.J., Pickett C.K., Weil J.V., Zwillich C.W. (1983). Sexual influence on the control of breathing. J. Appl. Physiol..

[11] Frappaolo A.M., Linder A.H., Wen T., Andrikopoulou M., Booker W.A., D’Alton M.E., Friedman A.M. (2023). Trends in and outcomes associated with obstructive sleep apnea during deliveries in the United States, 2000–2019. Am. J. Obstet. Gynecol. MFM.

[12] Tong X., Yang L., Jiang C., Weng Z., Zu A., Hou Y., Fang Y., Yang W., Sun S. (2023). A Review of the Associations between Obstructive Sleep Apnea and Gestational Diabetes Mellitus and Possible Mechanisms of Disease. Reprod. Sci..

[13] Song Y., Wang L., Zheng D., Zeng L., Wang Y. (2022). Sleep Disturbances Before Pregnancy and Subsequent Risk of Gestational Diabetes Mellitus. Nat. Sci. Sleep.

[14] Johns E.C., Hill E.A., Williams S., Sabil A., Riha R.L., Denison F.C., Reynolds R.M. (2022). High prevalence of obstructive sleep apnea in pregnant women with class III obesity: A prospective cohort study. J. Clin. Sleep Med..

[15] Izci Balserak B., Pien G.W., Prasad B., Mastrogiannis D., Park C., Quinn L.T., Herdegen J., Carley D.W. (2020). Obstructive Sleep Apnea Is Associated with Newly Diagnosed Gestational Diabetes Mellitus. Ann. Am. Thorac Soc..

[16] Zhang X., Zhang R., Cheng L., Wang Y., Ding X., Fu J., Dang J., Moore J., Li R. (2020). The effect of sleep impairment on gestational diabetes mellitus: A systematic review and meta-analysis of cohort studies. Sleep Med..

[17] Alonso-Fernández A., Cerdá Moncadas M., Álvarez Ruiz De Larrinaga A., Sánchez Barón A., Codina Marcet M., Rodríguez Rodríguez P., Gil Gómez A.V., Giménez Carrero M.P., Pía Martínez C., Cubero Marín J.P. (2021). Impact of Obstructive Sleep Apnea on Gestational Diabetes Mellitus. Arch. Bronconeumol..

[18] Jaimchariyatam N., Na-Rungsri K., Tungsanga S., Lertmaharit S., Lohsoonthorn V., Totienchai S. (2019). Obstructive sleep apnea as a risk factor for preeclampsia-eclampsia. Sleep Breath..

[19] Berry R.B., Budhiraja R., Gottlieb D.J., Gozal D., Iber C., Kapur V.K., Marcus C.L., Mehra R., Parthasarathy S., Quan S.F. (2012). Rules for scoring respiratory events in sleep: Update of the 2007 AASM Manual for the Scoring of Sleep and Associated Events. Deliberations of the Sleep Apnea Definitions Task Force of the American Academy of Sleep Medicine. J. Clin. Sleep Med..

[20] Chung F., Subramanyam R., Liao P., Sasaki E., Shapiro C., Sun Y. (2012). High STOP-Bang score indicates a high probability of obstructive sleep apnoea. Br. J. Anaesth..

[21] Johns M.W. (1991). A new method for measuring daytime sleepiness: The Epworth sleepiness scale. Sleep.

[22] Netzer N.C., Stoohs R.A., Netzer C.M., Clark K., Strohl K.P. (1999). Using the Berlin Questionnaire to identify patients at risk for the sleep apnea syndrome. Ann. Intern. Med..

[23] O’Brien L.M., Levine R.S., Dunietz G.L. (2021). The Berlin Questionnaire in pregnancy predominantly identifies obesity. J. Clin. Sleep Med..

[24] Bajaj S., Rice A.L., White P., Wiedmer A.M., Jacobson N.M., Jones N.R., Bazalakova M.H., Antony K.M. (2023). Clinical application of a previously validated pregnancy-specific screening tool for sleep apnea in a cohort with a high prevalence of obesity. Sleep Med. X.

[25] Seybold D.J., Bracero L.A., Power P., Koenig Z.A., Calhoun B.C., Bush S. (2022). Predicting perinatal outcomes with an obstructive sleep apnea screening tool. J. Med. Screen.

[26] Pearson F., Batterham A.M., Cope S. (2019). The STOP-Bang Questionnaire as a Screening Tool for Obstructive Sleep Apnea in Pregnancy. J. Clin. Sleep Med..

[27] Balserak B.I., Zhu B., Grandner M.A., Jackson N., Pien G.W. (2019). Obstructive sleep apnea in pregnancy: Performance of a rapid screening tool. Sleep Breath..

[28] Liu L., Su G., Wang S., Zhu B. (2019). The prevalence of obstructive sleep apnea and its association with pregnancy-related health outcomes: A systematic review and meta-analysis. Sleep Breath..

[29] Ghesquière L., Deruelle P., Ramdane Y., Garabedian C., Charley-Monaca C., Dalmas A.F. (2020). Obstructive sleep apnea in obese pregnant women: A prospective study. PLoS ONE.

[30] Kalkhoff S.M., Lutgendorf M.A., Morrison T.C., Han T., Spence D.L. (2022). A randomized controlled trial of sleep study surveillance with targeted autoregulated positive airway pressure therapy for obstructive sleep apnea in pregnancy. Am. J. Obstet. Gynecol. MFM.

[31] Schwartz J.E., Kovach A., Meyer J., McConnell C., Iwamoto H.S. (1998). Brief, intermittent hypoxia restricts fetal growth in Sprague-Dawley rats. Biol. Neonate.

[32] Chen L., Zadi Z.H., Zhang J., Scharf S.M., Pae E.K. (2018). Intermittent hypoxia in utero damages postnatal growth and cardiovascular function in rats. J. Appl. Physiol..

[33] Almendros I., Martínez-Ros P., Farré N., Rubio-Zaragoza M., Torres M., Gutiérrez-Bautista Á.J., Carrillo-Poveda J.M., Sopena-Juncosa J.J., Gozal D., Gonzalez-Bulnes A. (2019). Placental oxygen transfer reduces hypoxia-reoxygenation swings in fetal blood in a sheep model of gestational sleep apnea. J. Appl. Physiol..

[34] Keshavarzi F., Mehdizadeh S., Khazaie H., Ghadami M.R. (2018). Objective assessment of obstructive sleep apnea in normal pregnant and preeclamptic women. Hypertens. Pregnancy.

[35] Gumina D.L., Su E.J. (2023). Mechanistic insights into the development of severe fetal growth restriction. Clin. Sci..

[36] Kidron D., Bar-Lev Y., Tsarfaty I., Many A., Tauman R. (2019). The effect of maternal obstructive sleep apnea on the placenta. Sleep.

[37] Page M.J., McKenzie J.E., Bossuyt P.M., Boutron I., Hoffmann T.C., Mulrow C.D., Shamseer L., Tetzlaff J.M., Akl E.A., Brennan S.E. (2021). The PRISMA 2020 statement: An updated guideline for reporting systematic reviews. BMJ.

[38] Wells G., Shea B., O’Connell D., Peterson J., Welch V., Losos M., Tugwell P. (2014). Newcastle-Ottawa Quality Assessment Scale Cohort Studies.

[39] Wilson D.L., Fung A.M., Pell G., Skrzypek H., Barnes M., Bourjeily G., Walker S.P., Howard M.E. (2022). Polysomnographic analysis of maternal sleep position and its relationship to pregnancy complications and sleep-disordered breathing. Sleep.

[40] Hawkins M., Parker C.B., Redline S., Larkin J.C., Zee P.P., Grobman W.A., Silver R.M., Louis J.M., Pien G.W., Basner R.C. (2021). Objectively assessed sleep-disordered breathing during pregnancy and infant birthweight. Sleep Med..

[41] Wilson D.L., Howard M.E., Fung A.M., O’Donoghue F.J., Barnes M., Lappas M., Walker S.P. (2020). The presence of coexisting sleep-disordered breathing among women with hypertensive disorders of pregnancy does not worsen perinatal outcome. PLoS ONE.

[42] Suri J., Suri J.C., Arora R., Gupta M., Adhikari T. (2019). The Impact of Sleep-Disordered Breathing on Severity of Pregnancy-Induced Hypertension and Feto-Maternal Outcomes. J. Obstet. Gynecol. India.

[43] Telerant A., Dunietz G.L., Many A., Tauman R. (2018). Mild Maternal Obstructive Sleep Apnea in Non-obese Pregnant Women and Accelerated Fetal Growth. Sci. Rep..

[44] Kneitel A.W., Treadwell M.C., O’Brien L.M. (2018). Effects of maternal obstructive sleep apnea on fetal growth: A case-control study. J. Perinatol..

[45] Pamidi S., Marc I., Simoneau G., Lavigne L., Olha A., Benedetti A., Sériès F., Fraser W., Audibert F., Bujold E. (2016). Maternal sleep-disordered breathing and the risk of delivering small for gestational age infants: A prospective cohort study. Thorax.

[46] Bin Y.S., Cistulli P.A., Ford J.B. (2016). Population-Based Study of Sleep Apnea in Pregnancy and Maternal and Infant Outcomes. J. Clin. Sleep Med..

[47] Louis J.M., Mogos M.F., Salemi J.L., Redline S., Salihu H.M. (2014). Obstructive sleep apnea and severe maternal-infant morbidity/mortality in the United States, 1998–2009. Sleep.

[48] Facco F.L., Ouyang D.W., Zee P.C., Strohl A.E., Gonzalez A.B., Lim C., Grobman W.A. (2014). Implications of sleep-disordered breathing in pregnancy. Am. J. Obstet. Gynecol..

[49] Antony K.M., Agrawal A., Arndt M.E., Murphy A.M., Alapat P.M., Guntupalli K.K., Aagaard K.M. (2014). Association of adverse perinatal outcomes with screening measures of obstructive sleep apnea. J. Perinatol..

[50] Fung A.M., Wilson D.L., Lappas M., Howard M., Barnes M., O’Donoghue F., Tong S., Esdale H., Fleming G., Walker S.P. (2013). Effects of Maternal Obstructive Sleep Apnoea on Fetal Growth: A Prospective Cohort Study. PLoS ONE.

[51] Louis J., Auckley D., Miladinovic B., Shepherd A., Mencin P., Kumar D., Mercer B., Redline S. (2012). Perinatal outcomes associated with obstructive sleep apnea in obese pregnant women. Obstet. Gynecol..

[52] Chen Y.H., Kang J.H., Lin C.C., Wang I.T., Keller J.J., Lin H.C. (2012). Obstructive sleep apnea and the risk of adverse pregnancy outcomes. Am. J. Obstet. Gynecol..

[53] Louis J.M., Auckley D., Sokol R.J., Mercer B.M. (2010). Maternal and neonatal morbidities associated with obstructive sleep apnea complicating pregnancy. Am. J. Obstet. Gynecol..

[54] Yin T.T., Williams N., Burton C., Ong S.S., Loughna P., Britton J.R., Thornton J.G. (2008). Hypertension, fetal growth restriction and obstructive sleep apnoea in pregnancy. Eur. J. Obstet. Gynecol. Reprod. Biol..

[55] Sahin F.K., Koken G., Cosar E., Saylan F., Fidan F., Yilmazer M., Unlu M. (2008). Obstructive sleep apnea in pregnancy and fetal outcome. Int. J. Gynaecol. Obstet..

[56] Köken G., Sahin F.K., Cosar E., Saylan F., Yilmaz N., Altuntas I., Fidan F., Unlu M., Yilmazer M. (2007). Oxidative stress markers in pregnant women who snore and fetal outcome: A case control study. Acta Obstet. Gynecol. Scand..

[57] Johns M.W. (2000). Sensitivity and specificity of the multiple sleep latency test (MSLT), the maintenance of wakefulness test and the epworth sleepiness scale: Failure of the MSLT as a gold standard. J. Sleep Res..

[58] Rosenthal L.D., Dolan D.C. (2008). The Epworth sleepiness scale in the identification of obstructive sleep apnea. J. Nerv. Ment. Dis..

[59] Dunietz G.L., Sever O., DeRowe A., Tauman R. (2020). Sleep position and breathing in late pregnancy and perinatal outcomes. J. Clin. Sleep Med..

[60] Kember A.J., Scott H.M., O’Brien L.M., Borazjani A., Butler M.B., Wells J.H., Isaac A., Chu K., Coleman J., Morrison D.L. (2018). Modifying maternal sleep position in the third trimester of pregnancy with positional therapy: A randomised pilot trial. BMJ Open.

[61] Farabi S.S., Barbour L.A., Hernandez T.L. (2021). Sleep-disordered breathing in pregnancy: A developmental origin of offspring obesity?. J. Dev. Orig. Health Dis..

[62] Dave F., Cole S., Rees M. (2019). Obstructive sleep apnoea in multiple pregnancy. Aust. N. Z. J. Obstet. Gynaecol..

[63] Ryu G., Kim Y.M., Lee K.E., Choi S.J., Hong S.D., Jung Y.G., Oh S.Y., Kim H.Y. (2023). Obstructive Sleep Apnea Is Associated With Late-Onset Preeclampsia in Overweight Pregnant Women in Korea. J. Korean Med. Sci..

[64] Tayade S., Toshniwal S. (2022). Obstructive Sleep Apnea in Pregnancy: A Narrative Review. Cureus.

[65] Sanapo L., Bublitz M.H., Bai A., Mehta N., Messerlian G.M., Catalano P., Bourjeily G. (2022). Association between sleep disordered breathing in early pregnancy and glucose metabolism. Sleep.

[66] Dominguez J.E., Grotegut C.A., Wright M.C., Habib A.S. (2023). Obstructive Sleep Apnea among Gravidas with Chronic Hypertension Compared to Matched Controls: A Prospective Cohort Study. Anesth. Analg..

[67] Dominguez J.E., Habib A.S. (2022). Obstructive sleep apnea in pregnant women. Int. Anesthesiol. Clin..

[68] Joseph N., Shreeshaina, Loliem S.S.B., Gundi V.K., Subramanya M.B.H., Shashidhar S.B. (2020). An assessment of risks associated with obstructive sleep apnea and its relationship with adverse health outcomes among pregnant women. A multi-hospital based study. Adv. Respir. Med..

[69] Izci-Balserak B., Zhu B., Gurubhagavatula I., Keenan B.T., Pien G.W. (2019). A Screening Algorithm for Obstructive Sleep Apnea in Pregnancy. Ann. Am. Thorac Soc..

[70] Bourjeily G., Chambers A., Salameh M., Bublitz M.H., Kaur A., Coppa A., Risica P., Lambert-Messerlian G. (2019). Anthropometric Measures and Prediction of Maternal Sleep-Disordered Breathing. J. Clin. Sleep Med..

[71] Dominguez J.E., Grotegut C.A., Cooter M., Krystal A.D., Habib A.S. (2018). Screening extremely obese pregnant women for obstructive sleep apnea. Am. J. Obstet. Gynecol..

[72] Aguiar T., Montenegro N., Ferraz T. (2019). Screening tools for obstructive sleep apnea in extremely obese women during pregnancy. Am. J. Obstet. Gynecol..

[73] Izci-Balserak B., Keenan B.T., Corbitt C., Staley B., Perlis M., Pien G.W. (2018). Changes in Sleep Characteristics and Breathing Parameters During Sleep in Early and Late Pregnancy. J. Clin. Sleep Med..

[74] Sanapo L., Goldman D., Bourjeily G. (2021). Obstructive sleep apnea in pregnancy: 1 sleep study may not be enough in high-risk women. J. Clin. Sleep Med..

[75] Antony K.M., Jacobson N.M., Rice L., Wiedmer A.M., Mourey H., Bazalakova M.H. (2021). Obstructive Sleep Apnea in Pregnancy: Early Lessons from Our Sleep Pregnancy Clinic. WMJ.

[76] Stanek A., Brożyna-Tkaczyk K., Myśliński W. (2021). Oxidative Stress Markers among Obstructive Sleep Apnea Patients. Oxid. Med. Cell. Longev..

[77] Nugent R., Wee A., Kearney L., de Costa C. (2023). The effectiveness of continuous positive airway pressure for treating obstructive sleep apnoea in pregnancy: A systematic review. Aust. N. Z. J. Obstet. Gynaecol..

[78] Stajić D., Ilić D., Vuković J., Baturan B., Ilić A., Milovančev A. (2022). The effect of continuous positive airway pressure treatment on hypertensive disorder in pregnant women with obstructive sleep apnea. Sleep Breath..

[79] Rice A.L., Bajaj S., Wiedmer A.M., Jacobson N., Stanic A.K., Antony K.M., Bazalakova M.H. (2023). Continuous positive airway pressure treatment of obstructive sleep apnea and hypertensive complications in high-risk pregnancy. Sleep Breath..

[80] Migueis D.P., Urel A., Dos Santos C.C., Accetta A., Burla M. (2022). The cardiovascular, metabolic, fetal and neonatal effects of CPAP use in pregnant women: A systematic review. Sleep Sci..

[81] Kim M.S., Moon M.J., Lee Y.H., Chae K.Y., Ahn E.H. (2020). Treatment of superimposed preeclampsia on chronic hypertension in a twin pregnancy with automatic continuous positive airway pressure: A case report. BMC Pulm. Med..

[82] Chirakalwasan N., Amnakkittikul S., Wanitcharoenkul E., Charoensri S., Saetung S., Chanprasertyothin S., Chailurkit L.O., Panburana P., Bumrungphuet S., Thakkinstian A. (2018). Continuous Positive Airway Pressure Therapy in Gestational Diabetes With Obstructive Sleep Apnea: A Randomized Controlled Trial. J. Clin. Sleep Med..

[83] Huynh N., Drouin-Gagné L., Gilbert C., Arcache J.P., Rompré P., Morency A.M., Gagnon R., Kimoff J., Pamidi S. (2023). Adherence and efficacy of mandibular advancement splint treatment of sleep-disordered breathing during pregnancy: A pilot study. Sleep Breath..

