# Supplementary material for: A Relation between Obstructive Sleep Apnea in Pregnancy and Delivering Small for Gestational Age Infant—A Systematic Review
Source: J Clin Med. 2023 Sep 14;12(18):5972. doi: 10.3390/jcm12185972 (PMC10532405; doi:10.3390/jcm12185972)
Supplement: Supplementary file 1 [file jcm-12-05972-s001.zip › jcm-2520030-supplementary.pdf]

Table S1. Search strategy.

Database	Number of results	Search strategy
**PubMed**	255	(("Sleep Apnea, Obstructive" [Mesh]) OR (sleep AND apnea*) OR (sleep AND hypopnea*) OR (Upper AND Airway AND Resistance) OR (OSAHS) OR (OSA)) AND (("Fetal Growth Retardation" [Mesh]) OR (IUGR) OR (SGA) OR (FGR) OR (intrauterine AND growth AND (restrict* OR retard*)) OR (fetal AND growth AND (restrict* OR retard*)) OR ("Infant, Small for Gestational Age" [Mesh]) OR (small AND for AND gestational AND age) OR ("Birth Weight" [Mesh]) OR (birthweight*) OR (birth* AND weight*))
**Scopus**	444	TITLE-ABS-KEY (((sleep AND apnea*) OR (sleep AND hypopnea*) OR (Upper AND Airway AND Resistance) OR (OSAHS) OR (OSA)) AND ((IUGR) OR (SGA) OR (FGR) OR (intrauterine AND growth AND (restrict* OR retard*)) OR (fetal AND growth AND (restrict* OR retard*)) OR (small AND for AND gestational AND age) OR (birthweight*) OR (birth* AND weight*))) AND (LIMIT-TO (LANGUAGE, "English"))
**Cochrane**	45	#1 "Sleep Apnea, Obstructive"[Mesh]<br>#2 (sleep AND apnea*) OR (sleep AND hypopnea*) OR (Upper AND Airway AND Resistance) OR (OSAHS) OR (OSA)<br>#3 "Fetal Growth Retardation" [Mesh]<br>#4 (IUGR) OR (SGA) OR (FGR) OR (intrauterine AND growth AND (restrict* OR retard*)) OR (fetal AND growth AND (restrict* OR retard*))<br>#5 "Infant, Small for Gestational Age" [Mesh]<br>#6 (early AND for AND gestational AND age)<br>#7 "Birth Weight"[Mesh]<br>#8 (birthweight*) OR (birth* AND weight*)<br>• TRIALS
	744	

Table S2. Risk of bias assessed using the Newcastle-Ottawa quality assessment scale for cohort and case-control studies. A total score of 0-3 was considered unsatisfactory, 4-5 points satisfactory, 6-7 13 points good, and 8-9 points very good.

Study	**Selection** (max score of 4)	**Comparability** (max score of 2)	**Outcome** (max score of 3)	**Total score** (max score of 9)
Wilson et al. (2022) [39]	4	2	3	9
Hawkins et al. (2021) [40]	4	2	2	8
Wilson et al. (2020) [41]	3	1	3	7
Suri et al. (2019) [42]	4	2	2	8
Telerant et al. (2018) [43]	4	2	1	7
Kneitel et al. (2018) [44]	3	2	2	7
Pamidi et al. (2016) [45]	4	2	3	9
Bin et al. (2016) [46]	4	2	2	8
Louis et al. (2014) [47]	4	2	2	8
Facco et al. (2014) [48]	3	2	2	7
Antony et al. (2014) [49]	4	1	2	7
Fung et al. (2013) [50]	4	1	2	7
Louis et al. (2012) [51]	4	1	2	7
Chen et al. (2012) [52]	4	1	1	6
Louis et al. (2010) [53]	3	2	2	7

Yin et al. (2008) [54]	3	2	1	6
Sahin et al. (2007) [55]	3	1	2	6
Kőken et al. (2007) [56]	4	2	2	8
